# Impact of adding r-LH to r-FSH during ovarian stimulation on reproductive outcomes of women with polyendocrine metabolic ovarian syndrome undergoing *in vitro* fertilization

**DOI:** 10.3389/fendo.2026.1909206

**Published:** 2026-09-21

**Authors:** Inês Azevedo, Mafalda V. Moreira, Sofia S. Pereira, Carla Leal, Márcia Barreiro, Mariana P. Monteiro, Emídio Vale-Fernandes

**Affiliations:** 1 Unit for Multidisciplinary Research in Biomedicine (UMIB), School of Medicine and Biomedical Sciences (ICBAS), University of Porto, Porto, Portugal; 2 i3S - Instituto de Investigação e Inovação em Saúde, Universidade do Porto, Porto, Portugal; 3 ITR - Laboratory for Integrative and Translational Research in Population Health, Porto, Portugal; 4 Centre for Medically Assisted Procreation/Public Gamete Bank, Gynecology Department, Centro Materno-Infantil do Norte Dr. Albino Aroso (CMIN), Centro Hospitalar Universitário de Santo António (CHUdSA), Unidade Local de Saúde de Santo António (ULSSA), Porto, Portugal

**Keywords:** controlled ovarian stimulation, *in vitro* fertilization, polyendocrine metabolic ovarian syndrome, recombinant follicle-stimulating hormone, recombinant luteinizing hormone, reproductive outcomes

## Abstract

**Background:**

Polyendocrine Metabolic Ovarian Syndrome (PMOS) is a common endocrine disorder and a leading cause of anovulatory infertility. In women with PMOS, controlled ovarian stimulation (COS) often results in heterogeneous responses, with many oocytes of poor quality and lower fertilization rates. The combined use of recombinant Luteinizing Hormone (r-LH) and recombinant Follicle-Stimulating Hormone (r-FSH) has shown potential to improve Assisted Reproductive Technology (ART) outcomes in specific populations, such as women with hypogonadotropic hypogonadism or advanced age. However, its benefits in women with PMOS remain uncertain. This retrospective cohort study explores whether adding r-LH to r-FSH during COS improves reproductive outcomes in women with PMOS undergoing ART.

**Results:**

The study included 156 women diagnosed with PMOS according to Rotterdam criteria, undergoing COS at a single tertiary center. After propensity score matching for age, body mass index (BMI), anti-Müllerian hormone (AMH), LH, and LH/FSH ratio, 106 women were divided into two groups: 53 received r-LH/r-FSH, and 53 received r-FSH alone. Baseline data, stimulation parameters, and ART outcomes were analyzed. Women in the r-LH/r-FSH group required a higher cumulative FSH dose (2932 ± 848 vs. 2455 ± 588IU, p<0.002), produced more follicles on trigger day (13.91 ± 7.45 vs. 10.09 ± 5.55, p=0.014), and had more oocytes injected (13.81 ± 8.01 vs. 11.66 ± 5.57, p=0.0499). Multivariate analysis confirmed r-LH/r-FSH was independently associated with increased follicular recruitment, regardless of AMH levels. However, stimulation protocol was not a significant predictor of oocyte count; age and AMH remained the primary factors. Total FSH dose was not associated with follicle number. No significant differences were observed in cumulative live birth rate; however, clinical pregnancy rate (CPR) after frozen embryo transfer (FET) was higher in the control group (54.5% vs. 25.0%, p=0.043).

**Conclusion:**

Adding r-LH to r-FSH during COS in women with PMOS was associated with increased follicular recruitment but did not improve live birth or overall pregnancy rates. Notably, CPR after FET was lower with r-LH/r-FSH. These findings suggest r-LH may modulate ovarian response, but further research is needed to clarify its role in ART protocols. Due to the retrospective, non-randomized design, these results are hypothesis-generating, and routine use of r-LH in this setting cannot currently be recommended.

## 1 Introduction

Polyendocrine Metabolic Ovarian Syndrome (PMOS) is recognized as the most prevalent endocrine disorder in women of reproductive age, with a global prevalence estimated between 10-13% according to Rotterdam Criteria (1). PMOS stands out as a leading cause of anovulatory infertility, being responsible for over 80% of such cases (2). When pregnancy occurs, women with PMOS face a heightened risk of obstetric complications, such as miscarriage, gestational diabetes, pregnancy-induced hypertension, and preterm delivery (3, 4). Furthermore, the impact of PMOS can extend beyond reproductive health, as women affected often present metabolic comorbidities, such as obesity and insulin resistance, alongside increased long-term risk of cardiovascular disease and type 2 diabetes (5).

Management of PMOS-related infertility follows a stepwise approach depending on the woman´s phenotype. In women with overweight or obesity, weight loss should be prioritized through lifestyle modifications, pharmacotherapy, or bariatric surgery, as appropriate (2, 6). When these measures are insufficient, pharmacological ovulation induction is indicated (2, 6). Whereas for women with advanced maternal age, in whom previous interventions failed, or for couples with additional infertility factors, Assisted Reproductive Technology (ART) is then proposed (2), requiring controlled ovarian stimulation (COS) with Follicle-Stimulating Hormone (FSH) to recruit follicles and retrieve oocytes for *in vitro* fertilization (IVF) or intracytoplasmic sperm injection (ICSI). However, in women with PMOS, ovarian response to COS can be widely variable, typically resulting in a high number of follicles recruited and oocytes retrieved, although quality is often compromised (7, 8). Moreover, the dysfunctional follicular microenvironment and oocyte immaturity often lead to lower fertilization rates and a higher incidence of miscarriages (4, 7, 8).

The combined use of recombinant Luteinizing Hormone and recombinant Follicle-Stimulating Hormone (r-LH/r-FSH) in COS has been introduced to improve oocyte maturation and embryo quality, thereby enhancing implantation and clinical pregnancy potential in women with hypogonadotropic hypogonadism (LH and FSH deficiency) (9, 10). More recently, this approach has also been proposed for women with advanced maternal age (over 35 years) and hypo-responders to r-FSH monotherapy (11–13). Furthermore, the use of r-LH/r-FSH for COS in women with PMOS has also been suggested to enhance follicular recruitment and oocyte quality by optimizing follicle-stimulating pathways, and potentially improving IVF outcomes, based on the rationale that LH and FSH exert complementary actions in supporting folliculogenesis (14, 15).

However, the supporting evidence on the use of r-LH/r-FSH for COS in women with PMOS remains limited and inconsistent, lacking further evidence to contribute to clarifying the ongoing controversy. On one hand, a systematic review and meta-analysis including nine studies using a gonadotropin-releasing hormone (GnRH) antagonist protocol reported that r-LH/r-FSH improved follicular recruitment and oocyte yield compared to r-FSH alone (16). On the other hand, a retrospective study specifically examining women with PMOS undergoing GnRH antagonist protocols found that LH supplementation was associated with impaired cumulative live birth rates (CLBR) despite reducing FSH requirements and shortening stimulation duration (17). Similarly, in broader IVF populations, a large retrospective analysis of 1,470 women found significantly lower CLBR with r-FSH/r-LH compared to r-FSH alone in unselected patients, although outcomes were comparable in poor and suboptimal responders (18). More recently, a large propensity score-matched study including over 2,000 embryos from IVF/Preimplantation Genetic Testing for Aneuploidy (PGT-A) cycles demonstrated that LH supplementation during ovarian stimulation did not improve embryo euploidy rates or reproductive outcomes following frozen euploid embryo transfer (19). This inconsistency in the literature underscores the need for further evidence to clarify the role of r-LH supplementation specifically in women with PMOS undergoing COS with a GnRH antagonist protocol.

Therefore, the present study aimed to explore whether the use of r-LH/r-FSH for COS results in improved reproductive outcomes compared to r-FSH alone in women with PMOS.

## 2 Materials and methods

### 2.1 Study design

This was a retrospective cohort study aimed to compare the reproductive outcomes of women with PMOS undergoing COS with r-LH/r-FSH as compared to r-FSH alone. This study was conducted at a single public academic tertiary center for Medically Assisted Reproduction and granted institutional approval by the local Ethics Committee under the registration number 2025-278(236-CAC/236-CE). Data for analysis was retrieved from an anonymized database migrated from the women’s electronic medical records, and therefore, the need for consent has been waived.

### 2.2 Study population

The study enrolled all women diagnosed with PMOS according to the Rotterdam criteria (6) who underwent COS for ART (IVF or ICSI) between January 2021 and December 2024. A presumptive diagnosis of PMOS that did not meet the Rotterdam criteria, or the presence of clinical/biochemical HA due to causes other than PMOS, were considered exclusion criteria. Women with endometriosis, thyroid disease, and other endocrine disorders were also excluded.

From the initial cohort of 197 ICSI cycles and 114 IVF cycles, a total of 156 women deemed eligible were enrolled for data analysis accordingly to the inclusion and exclusion criteria. Therefore, exclusion was not driven by missing values (key variables used in the primary and secondary outcome analyses were complete for all included participants). After the propensity score matching (PSM), the study participants were divided into two groups according to the stimulation protocol used during COS: women who received combined r-LH/r-FSH (n=53) and women who received r-FSH alone, as controls (n=53).

### 2.3 Ovarian stimulation protocol

COS in our study was conducted using either r-LH/r-FSH in the intervention group, and r-FSH in the control group. All women underwent a GnRH antagonist protocol to suppress pituitary gonadotropin secretion, with gonadotropins administration starting on the 1st to 3rd day of the menstrual cycle. The trigger was administered using a GnRH agonist to mitigate the increased risk of ovarian hyperstimulation syndrome (OHSS) in women with PMOS. The trigger was given once more than three follicles measuring over 17-18mm were observed. The choice and initial dosage were based on age, ovarian reserve, and clinician judgment. In the intervention group, r-LH was administered daily using a fixed-dose combination of r-FSH and r-LH in a 2:1 ratio from the first day of COS until the trigger day. On the third day after oocyte fertilization, one or two embryos with the highest score were selected for transfer, while the remaining embryos were cryopreserved or allowed to develop until the blastocyst stage for fresh-embryo transfer or to be frozen for subsequent frozen embryo transfer (FET). The decision to freeze all embryos was adopted according to local clinical criteria, which included factors such as the risk of OHSS, unsuitable endometrial thickness, premature progesterone elevation, or personal circumstances that favored FET over fresh-embryo transfer cycle.

### 2.4 Data collection

Data regarding baseline clinical characteristics, ovarian stimulation parameters, and reproductive outcomes were obtained from the anonymized database for analysis.

Baseline clinical characteristics included maternal age, type and duration of infertility, body mass index (BMI), FSH, LH, LH/FSH ratio, anti-Müllerian hormone (AMH), estradiol, and progesterone levels. Cycle characteristics included total FSH dose, duration of stimulation, endometrial thickness on the trigger day and on the day of oocyte retrieval, number of follicles on trigger day, number of cumulus-oocyte complexes (COCs) obtained, mature oocytes and immature oocytes, oocytes injected (ICSI), two pronucleated oocytes (2PN), embryos on day 2 and day 3, transferred (fresh-embryo transfer) and cryopreserved embryos, number of FET cycles, embryos used, clinical pregnancies and live births.

### 2.5 Outcomes

The primary outcomes were CLBR and live birth rate (LBR). CLBR was defined as the number of women who achieved at least one live birth following all embryo transfers resulting from a single ovarian stimulation cycle, including both fresh and frozen embryo transfers, divided by the total number of women in each group. Secondary outcomes included clinical pregnancy rate (CPR), ovarian response, and embryo quality. Clinical pregnancy was defined as the visualization of one or more gestational sacs on ultrasonography. Additionally, CPR and LBR were analyzed separately for fresh embryo transfer and FET cycles. For fresh embryo transfer outcomes, only women who underwent fresh embryo transfer were included (Control: n=26; Intervention: n=19). For FET outcomes, only women who underwent at least one FET cycle were included (Control: n=22; Intervention: n=28).

### 2.6 Statistical analysis

Statistical analysis was performed using the Statistical Package for the Social Sciences, version 29 (IBM SPSS 29). PSM was performed to reduce potential confounding by measured covariates. Propensity scores were estimated using binary logistic regression, with treatment group as the dependent variable and age, BMI, AMH levels, LH levels, and the LH/FSH ratio as covariates. Matching was performed in a 1:1 ratio, using a caliper of 0.2 standard deviations of the logit of the propensity score. The logit was standardized using the mean and standard deviation of the pre-matching analysis sample, and a maximum absolute difference of 0.2 between standardized logit scores was permitted within each pair. Only complete matched pairs were retained for subsequent analyses. Categorical data were presented as percentages and the number of cases, while continuous data were reported as mean ± standard deviation (SD). Continuous variables were tested for normality using the Kolmogorov–Smirnov test. Normally distributed variables were compared using the independent-samples t-test, whereas non-normally distributed variables were analyzed using the Mann–Whitney U test. Categorical variables were compared using the chi-square test or Fisher’s exact test, as appropriate. Linear regression analysis was performed to assess whether stimulation protocol, total FSH dose, AMH, age, BMI and LH were independently associated with the number of follicles, the number of COCs, and the number of oocytes injected. Initially, a univariate analysis was conducted, in which each independent variable was tested individually against each of the dependent variables. Subsequently, a multivariate linear regression model was developed for each outcome (number of follicles, number of COCs, and number of oocytes injected), including variables with clinical relevance and/or statistical significance in the univariate analysis. The results are presented as β coefficients, 95% confidence intervals (95% CI), and *p*-values. Throughout the statistical analysis, a *p*-value < 0.05 was considered statistically significant.

## 3 Results

### 3.1 Comparison of baseline clinical and reproductive characteristics between the r-LH/r-FSH and r-FSH monotherapy

The clinical features of women with PMOS who underwent COS either with r-LH/r-FSH or r-FSH monotherapy are presented in **Table 1**. The baseline clinical and reproductive characteristics of the two groups of women did not differ in any parameter evaluated, including duration of infertility, FSH, estradiol or progesterone levels.

**Table 1:** Baseline clinical and reproductive characteristics of women with Polyendocrine Metabolic Ovarian Syndrome (PMOS) who underwent controlled ovarian stimulation (COS) with recombinant Follicle-Stimulating Hormone (r-FSH) or recombinant Luteinizing Hormone/recombinant Follicle-Stimulating Hormone (r-LH/r-FSH).

Variable		Control (r-FSH) n = 53	Intervention (r-LH + r-FSH) n = 53	*p* – value
		Mean ± SD	Mean ± SD	
Age (years)		33.44 ± 3.74	33.41 ± 3.76	NS (0.979)
Duration of infertility (months)		51.19 ± 24.35	44.19 ± 25.61	NS (0.698)
BMI (kg/m^2^)		26.08 ± 4.56	25.08 ± 4.33	NS (0.440)
FSH (mIU/mL)		6.22 ± 1.74	6.36 ± 1.44	NS (0.742)
LH (mIU/mL)		8.92 ± 3.68	8.20 ± 3.26	NS (0.598)
LH/FSH ratio		1.48 ± 0.60	1.35 ± 0.61	NS (0.345)
Estradiol (pg/mL)		75.25 ± 67.52	52.01 ± 33.61	NS (0.350)
Progesterone (ng/mL)		1.50 ± 1.53	3.29 ± 5.21	NS (0.064)
AMH (ng/mL)		4.47 ± 2.51	5.12 ± 3.87	NS (0.867)
Type of infertility				
Primary		43 (81.10%)	46 (86.80%)	NS (0.427)^a^
Secondary		10 (18.90%)	7 (13.20%)	

BMI, Body Mass Index; FSH, Follicle-Stimulating Hormone; IU, International Units; LH, Luteinizing Hormone; AMH, anti-Müllerian hormone; NS, Not Statistically Different. ^a^Chi-Square Test.

### 3.2 Comparison of cycle characteristics and reproductive outcomes between r-LH/r-FSH and r-FSH monotherapy

Cycle characteristics and reproductive outcomes are summarized in **Table 2**. The distribution of ART technique between groups was significantly different (*p* = 0.025) with a higher proportion of IVF cycles in the intervention group (45.30%) compared to the control group (24.50%). Women receiving r-LH/r-FSH required a higher cumulative FSH dose (2455 ± 718.14 vs 2932.03 ± 570.19 mIU/mL in r-LH/r-FSH vs control, *p* < 0.002) and exhibited a significantly higher follicle count on trigger day (10.09 ± 5.55 vs 13.91 ± 7.45 in control vs r-LH/r-FSH, *p* = 0.014). Additionally, the number of oocytes injected was significantly higher in the intervention group (11.66 ± 5.57 vs 13.81 ± 8.01 in control vs r-LH/r-FSH, *p* = 0.0499). No statistically significant differences were observed between the two groups in fertilization rate and CLBR. When stratified by transfer type (**Table 3**), no significant differences were observed between groups in CPR or LBR for fresh embryo transfer cycles (30.8% vs 36.8%, *p* = 0.754; 26.9% vs 21.1%, *p* = 0.736, respectively). On the other hand, significant differences were observed when comparing CPR for FET between the two groups, with a higher CPR on the control group (54.5% vs 25.0% in control vs r-LH/r-FSH, *p* = 0.043). However, no significant differences were observed on LBR (18.2% vs 7.1% in control vs r-LH/r-FSH, *p* = 0.0385).

**Table 2:** Ovarian stimulation characteristics and Assisted Reproductive Technology (ART) outcomes.

Variable		Control (r-FSH) N = 53	Intervention (r-LH/r-FSH) N = 53	*p* - value
		Mean ± SD	Mean ± SD	
ART technique [n (%)]				
IVF		**13 (24.50%)**	**24 (45.30%)**	**0.025^a^**
ICSI		**40 (75.50%)**	**29 (54.70%)**	
Total FSH dose (IU)		**2455.47 ± 587.67**	**2932.03 ± 848.39**	**< 0.002**
Duration of stimulation (days)		10.47 ± 1.81	10.75 ± 2.26	NS (0.725)
Endometrial thickness on the trigger day		9.72 ± 1.92	9.35 ± 1.61	NS (0.289)
Endometrial thickness on the day of follicular puncture		10.23 ± 2.30	9.91 ± 1.84	NS (0.456)
No. of follicles		**10.09 ± 5.55**	**13.91 ± 7.45**	**0.014**
No. of COCs		14.91 ± 5.52	15.34 ± 8.33	NS (0.498)
No. of oocytes injected		**11.66 ± 5.57**	**13.81 ± 8.01**	**0.0499**
No. of mature oocytes		11.75 ± 4.73	12.53 ± 7.44	NS (0.209)
No. of immature oocytes		2.91 ± 1.28	2.59 ± 2.15	NS (0.081)
Oocyte immaturity rate (%)		20.16 ± 8.58	18.59 ± 13.91	NS (0.079)
No. of two pronucleated oocytes (2PN)		6.75 ± 4.08	7.38 ± 5.52	NS (0.327)
Fertilization rate (%)		43.72 ± 17.87	45.81 ± 15.54	NS (0.630)
Cleavage rate (%)		98.91 ± 3.91	99.25 ± 3.58	NS (0.638)
No. of embryos day 2		4.13 ± 3.48	4.19 ± 4.20	NS (0.570)
No. of obtained embryos (day 3)		3.78 ± 2.93	3.34 ± 2.77	NS (0.500)
Fresh-embryo transfer [n (%)]		26 (49.1%)	19 (35.8%)	NS (0.169)^a^
No. of cryopreserved embryos		2.51 ± 2.24	3.19 ± 2.86	NS (0.349)
No. of freeze-all [n (%)]		22 (41.5%)	28 (52.8%)	NS (0.243)^a^
No. of embryos used		2.88 ± 2.15	3.19 ± 2.55	NS (0.349)
Cumulative Live Birth Rate (CLBR) [n (%)]		11 (20.8%)	6 (11.3%)	NS (0.290)^b^

Significant differences are highlighted in bold.

ART, Assisted Reproductive Technology; IVF, *In Vitro* Fertilization; ICSI, Intracytoplasmic Sperm Injection; FSH, Follicle-stimulating Hormone; IU, International Units; COCs, Cumulus–Oocyte Complexes; 2PN, two pronucleated oocytes; CLBR, Cumulative Live Birth Rate; NS, not statistically different. ^a^Chi-Square Test; ^b^Fisher’s Exact Test.

**Table 3:** Clinical Pregnancy Rate (CPR) and Live Birth Rate (LBR) according to transfer type (fresh vs. frozen).

Variable	Control (r-FSH) N = 26	Intervention (r-LH/r-FSH) N = 19	*p* - value
	Mean ± SD	Mean ± SD	
Fresh Embryo Transfer Clinical Pregnancy Rate (CPR) [n (%)]	8 (30.8%)	7 (36.8%)	NS (0.754)^b^
Fresh Embryo Transfer Live Birth Rate (LBR) [n (%)]	7 (26.9%)	4 (21.1%)	NS (0.736)^b^

Variable	Control (r-FSH) N = 22	Intervention (r-LH/r-FSH) N = 28	*p* - value
	Mean ± SD	Mean ± SD	
Frozen Embryo Transfer (FET) Clinical Pregnancy Rate (CPR) [n (%)]	**12 (54.5%)**	**7 (25.0%)**	**0.043^b^**
Frozen Embryo Transfer (FET) Live Birth Rate (LBR) [n (%)]	4 (18.2%)	2 (7.1%)	NS (0.385)^b^

Significant differences are highlighted in bold.

CPR, Clinical Pregnancy Rate; LBR, Live Birth Rate; FET, Frozen Embryo Transfer; NS, not statistically different. ^b^Fisher’s Exact Test.

### 3.3 Univariate and multivariate regression analysis of factors associated with follicular recruitment and oocyte yield

The results of the univariate and multivariate linear regression analyses are summarized in **Table 4**. In univariate linear regression analysis for the number of follicles, the combined protocol with r-LH/r-FSH was significantly associated with a higher number of follicles compared with r-FSH alone (β = 3.132; 95% CI: 0.648–5.616; *p* = 0.014), and AMH was also associated with the higher follicle count (β = 0.506; 95% CI: 0.175–0.945; *p* = 0.005). In contrast, total FSH dose (β = 0.045; 95% CI: -0.001–0.002; *p* = 0.644) was not associated with the higher follicle count. Age was negatively associated with the number of follicles (β = -0.675; 95% CI: -0.946– -0.405; *p* < 0.001), while BMI showed no significant association (β = -0.004; 95% CI: -0.235–0.227; *p* = 0.972), and LH was also not significantly associated (β = -0.169; 95% CI: -0.461–0.122; *p* = 0.252).

**Table 4:** Univariate and multivariate linear regression analyses of clinical and therapeutic factors associated with the number of recruited follicles and cumulus-oocyte complexes (COCs).

Variable	Number of follicles		Number of COCs		No. of oocytes injected	
	Univariate analysis β (95% CI)	Multivariate analysis β (95% CI)	Univariate analysis β (95% CI)	Multivariate analysis β (95% CI)	Univariate analysis β (95% CI)	Multivariate analysis β (95% CI)
Stimulation protocol (r-FSH only vs r-LH/r-FSH)	**3.132 (0.648 – 5.616)** ***p* = 0.014**	**3.500 (1.494 – 5.505)** ***p* < 0.001**	1.585 (-1.278 – 4.448) *p* = 0.275	**-**	1.885 (-0.494 – 4.263) *p* = 0.120	–
Total FSH dose (IU)	0.045 (-0.001 **–**0.002) *p* = 0.644	**-**	0.001 (-0.002 - 0.002) *p* = 0.802	–	0.001 (-0.001 – 0.003) *p* = 0.338	–
AMH (ng/mL)	**0.506 (0.175 – 0.945)** ***p* = 0.005**	**0.316 (0.094 – 0.538)** ***p* = 0.005**	**0.594 (0.158 – 1.029)** ***p* = 0.008**	**0.316 (0.042 – 0.590)** ***p* = 0.024**	**0.338 (0.080 – 0.595)** ***p* = 0.011**	**0.288 (0.036 – 0.540)** ***p* = 0.026**
LH (mIU/mL)	- 0.169 (-0.461 – 0.122) p = 0.252	**-**	0.104 (-0.239 – 0.447) *p* = 0.550	–	0.071 (-0.241 – 0.384) *p* = 0.654	–
Age (years)	**- 0.675 (-0.946 - - 0.405)** ***p* < 0.001**	**- 0.620 (- 0.880 - - 0.360)** ***p* < 0.001**	**- 0.645 (-0.971 - - 0.319)** ***p* < 0.001**	**- 0.599 (- 0.923 - - 0.275)** ***p* < 0.001**	**- 0.527 (- 0.827 - - 0.227)** ***p* < 0.001**	**- 0.485 (-0.784 - - 0.187)** ***p* = 0.002**
BMI (kg/m^2^)	-0.004 (-0.235 – 0.227) *p* = 0.972	**-**	- 0.095 (-0.366 – 0.175) *p* = 0.487	–	- 0.120 (-0.366 – 0.126) *p* = 0.338	–

Significant differences are highlighted in bold.

COCs, Cumulus-oocyte Complexes; r-FSH, recombinant Follicle-Stimulating Hormone; r-LH, recombinant Luteinizing Hormone; FSH, Follicle-Stimulating Hormone; IU, International Units; AMH, anti-Müllerian hormone; LH, Luteinizing Hormone; BMI, Body Mass Index.

In the multivariate analysis, AMH, age and stimulation protocol remained independently associated with follicle count. AMH retained a significant positive association (β = 0.316; 95% CI: 0.094–0.538; *p* = 0.005), as did stimulation protocol with r-LH/r-FSH compared with r-FSH alone (β = 3.500; 95% CI: 1.494–5.505; *p* < 0.001). Thus, AMH and treatment with r-LH/r-FSH were independently associated with a higher number of follicles. On the other hand, age was negatively associated with the follicle count (β = -0.620; 95% CI: -0.880– -0.360; *p* < 0.001).

In the univariate analysis for the number of COCs, stimulation protocol with r-LH/r-FSH (β = 1.585; 95% CI: −1.278–4.448; *p* = 0.275) and total FSH dose (β = 0.001; 95% CI: -0.002–0.002; *p* = 0.802) were not significantly associated with this outcome. However, AMH was associated with the number of COCs (β = 0.594; 95% CI: 0.158–1.029; *p* = 0.008). Age was also negatively associated with the number of COCs (β = -0.645; 95% CI: -0.971– -0.319; *p* < 0.001), while LH (β = 0.104; 95% CI: -0.239–0.447; *p* = 0.550) and BMI (β = -0.095; 95% CI: -0.366–0.175; *p* = 0.487) showed no significant association. For the number of COCs, multivariate analysis showed that AMH remained independently and positively associated (β = 0.316; 95% CI: 0.042–0.590; *p* = 0.024), while age remained independently and negatively associated (β = -0.599; 95% CI: -0.923– -0.275; *p* < 0.001).

Regarding the number of oocytes injected, in univariate analysis stimulation protocol (β = 1.885; 95% CI: -0.494–4.263; *p* = 0.120), total FSH dose (β = 0.001; 95% CI: -0.001–0.003; *p* = 0.338), LH (β = 0.071; 95% CI: -0.241–0.384; *p* = 0.654) and BMI (β = -0.120; 95% CI: -0.366–0.126; *p* = 0.338) were not significantly associated, whereas AMH (β = 0.338; 95% CI: 0.080–0.595; *p* = 0.011) and age (β = -0.527; 95% CI: -0.827– -0.227; *p* < 0.001) were significantly associated with this outcome. The multivariate analysis confirmed AMH as an independent positive predictor (β = 0.288; 95% CI: 0.036–0.540; *p* = 0.026) and age as an independent negative predictor (β = -0.485; 95% CI: -0.784– -0.187; *p* = 0.002).

## 4 Discussion

This retrospective study evaluated the reproductive outcomes of women with PMOS after COS with r-LH/r-FSH vs r-FSH alone. The results demonstrated that, in women with PMOS, COS with r-LH/r-FSH led to significantly increased follicular recruitment and a higher number of mature oocytes injected (ICSI) but did not improve CLBR, LBR or CPR compared with r-FSH alone. Moreover, multivariate analysis showed that the effect of r-LH/r-FSH on follicle recruitment was independent of baseline AMH levels.

A central hallmark of PMOS is the increased frequency of GnRH pulsatility, which preferentially drives LH secretion over FSH, leading to an elevated LH/FSH ratio (20–22). Excess LH overstimulates theca cells, leading to androgen overproduction, while relative FSH deficiency impairs aromatase activity, reducing androgen-to-estrogen conversion (21, 23). The resulting hyperandrogenism disrupts oocyte maturation and follicular development, impairing the selection and growth of a dominant follicle, leading to follicular arrest and anovulation (21). Nevertheless, previous studies report conflicting findings regarding the relationship between basal LH levels, oocyte quality, and pregnancy outcomes during IVF, demonstrating no significant effect of high basal LH levels on the IVF outcomes (24–26).

LH plays an important role in folliculogenesis and is known to operate within a strict concentration window; while a minimum threshold is essential for adequate steroidogenesis, concentrations exceeding the “ceiling effect” can impair pre-ovulatory development (15, 27, 28). In our study, women with PMOS underwent COS after endogenous LH suppression by GnRH antagonist, while r-LH and r-FSH were administered in physiological proportions, which increased follicular recruitment without jeopardizing oocyte maturation, as evidenced by comparable oocyte maturation rates between groups.

Given the characteristically high LH levels in PMOS, GnRH antagonists are commonly used as a strategy to suppress endogenous gonadotropin secretion (6, 29). However, despite mitigating the risk for OHSS, this approach has the drawback of inducing profound LH suppression, negatively impacting folliculogenesis, and oocyte maturation, thus compromising oocyte competence (30, 31). This adverse effect can potentially be overcome by using r-LH/r-FSH for COS. Indeed, a recent systematic review and meta-analysis, including nine studies in which women underwent IVF/ICSI using a GnRH antagonist protocol, demonstrated that r-LH/r-FSH improved follicular recruitment and oocyte yield (16). Our study results further suggest that this is still observed in women with PMOS, since r-LH/r-FSH optimized follicular recruitment during COS under GnRH antagonists. Moreover, similar findings were reported in a recent retrospective study comparing IVF reproductive outcomes in women with PMOS undergoing COS with r-LH/r-FSH under a GnRH antagonist protocol, reporting greater follicular recruitment with r-LH/r-FSH; however, the CLBR was lower compared with r-FSH alone (17). Although the mechanisms responsible for these effects are yet to be uncovered, the putative effect of LH on follicular fluid composition, which directly impacts oocyte competence, is a possible explanation (30, 32). Previous data suggest that r-LH during COS may modify the follicular fluid towards a more favorable profile by increasing progesterone and estrogen levels and reducing AMH concentrations (30, 32–34). However, in the present study the addiction of r-LH did not result in significant improvements in oocyte maturation rate, fertilization rate or CLBR, suggesting that the impact of r-LH on oocyte competence in PMOS may be more limited than in other populations.

In fact, the absence of improvement in oocyte maturation rate, fertilization rate and live birth outcomes despite enhanced follicular recruitment may reflect the inherent complexity of oocyte competence in PMOS, which extends beyond folliculogenesis alone. At the mitochondrial level, PMOS is associated with impaired oxidative phosphorylation and increased reactive oxygen species production in oocytes, leading to inadequate energy supply that may compromise oocyte quality independently of gonadotropin stimulation (22, 35). Furthermore, the potential impact of r-LH supplementation on embryo chromosomal status remains uncertain. A recent large propensity score-matched study including over 2,000 embryos from IVF/PGT-A cycles found no significant association between LH activity supplementation during ovarian stimulation and embryo euploidy rates (45.8% vs. 45.2%, p = 0.749), nor with CPR or LBR following frozen euploid embryo transfer (19). These findings of no differences in CPR and LBR are consistent with our results. Finally, endometrial receptivity represents an additional determinant of ART success that may be differentially affected in PMOS through mechanisms including altered endometrial gene expression, impaired progesterone signaling, increased testosterone levels and the metabolic comorbidities frequently associated with the syndrome, such as insulin resistance and obesity, none of which would be expected to be corrected by r-LH supplementation during ovarian stimulation (22, 36).

Indeed, ART success rates in PMOS are influenced by variables that transcend the ovarian response regardless of the stimulation protocol used, such as endometrial receptivity and metabolic profile, including obesity and insulin resistance. In the present study, age and BMI were comparable between the two groups, reducing the likelihood that differences in these characteristics accounted for the observed outcomes. Consistent with expected physiological trends, age was an independent negative predictor of follicle count, COCs number, and oocytes injected across both univariate and multivariate models, whereas BMI showed no significant association with any of these outcomes. Nevertheless, PMOS is a highly heterogeneous disorder, and BMI and age alone do not capture its full phenotypic spectrum. Differences in other characteristics, including androgen status and insulin resistance, may contribute to variability in response to ovarian stimulation and may influence the potential effect of r-LH supplementation.

Although the r-LH/r-FSH group received a significantly higher cumulative FSH dose, linear regression analysis demonstrated that total FSH dose was not significantly associated with follicle count (β = 0.045; 95% CI: −0.001–0.002; *p* = 0.644). These findings indicate that the higher cumulative FSH dose observed in the r-LH/r-FSH group did not independently drive follicular recruitment, supporting the interpretation that the observed difference in follicle count reflects the specific effect of r-LH supplementation rather than greater FSH exposure. Importantly, stimulation protocol remained independently associated with follicle count even after adjusting for total FSH dose, AMH, age, and BMI (multivariate β = 3.500; 95% CI: 1.494–5.505; *p* < 0.001), reinforcing that the observed increase in follicular recruitment is more likely to reflect the addition of r-LH rather than a dose-dependent FSH effect. The absence of differences in CLBR may also reflect the study’s limited sample size, which may lack the power to detect differences in such complex multifactorial outcomes.

A key finding in this study is that the impact of r-LH/r-FSH on follicle count was independent of baseline AMH levels. This is of particular interest in women with PMOS, who typically present with elevated serum AMH levels, which inhibits follicular recruitment, contributing to anovulation and infertility (37, 38). Indeed, elevated AMH levels in women with PMOS were shown to serve as a biomarker of disease severity and worse reproductive prognosis (37). Our study findings corroborate the synergistic effect of both gonadotropins and further suggest that the benefits of adding r-LH to r-FSH in COS are not restricted to women with hypogonadotropic hypogonadism and diminished ovarian reserve and are also observed in women with PMOS and high AMH levels. In turn, r-LH may further enhance follicular recruitment by driving the production of thecal androgens, which upregulate FSH receptor expression on granulosa cells (39). Consequently, the addition of r-LH may partially counterbalance the reduced follicular sensitivity to FSH associated with elevated AMH levels, potentially facilitating follicular recruitment and development.

It is also noteworthy that more than half of the women in the r-LH/r-FSH group (52.8%) underwent a freeze-all strategy, compared to 41.5% in the control group. This clinical decision is frequently driven by the estimated risk of OHSS, considering the more exuberant follicular response in women with PMOS (40). Since the freeze-all technique is associated with higher CLBR and a reduced risk of OHSS compared to fresh embryo transfers (40), the use of r-LH may indirectly lead to a higher rate of elective cryopreservation by optimizing follicular recruitment. Consequently, the potential differences in reproductive outcomes may only become evident after subsequent FET cycles. When pregnancy outcomes were analyzed separately for fresh and frozen embryo transfer cycles, CPR in FET cycles was higher in the control group (54.5% vs 25.0% in control vs r-LH/r-FSH, *p* = 0.043). Notwithstanding this finding, no differences in LBR between the groups were observed.

This study harbors several limitations that warrant careful consideration. First, its retrospective and observational nature. Additionally, this study did not include a prospective sample size calculation, as the cohort was defined retrospectively based on the number of eligible patients treated during the study period. Consequently, it cannot be determined whether the absence of significant differences in pregnancy and live birth rates reflects a true lack of clinical benefit or insufficient statistical power to detect a real effect. Second, the relatively small sample size limits the statistical power to identify differences in reproductive outcomes. Although subgroup analyses stratified by age, BMI, and AMH concentration were considered, the limited sample size would render these analyses statistically underpowered and potentially misleading. Such stratified analyses, ideally supported by an *a priori* sample size calculation, should be prioritized in future prospective randomized controlled trials to more robustly identify subpopulations that may derive greater benefit from r-LH supplementation. Third, although the primary and secondary outcomes were objective measures, residual confounding could not be entirely ruled out, including subtle variations in ovarian stimulation protocols and other unmeasured confounding factors. Embryo morphological grading, which may act as an additional confounder of CLBR given the higher freeze-all rate observed in the intervention group, was also not incorporated into the statistical adjustment, representing a further source of potential residual confounding. Likewise, androgen concentrations, fasting glucose levels, and history of recurrent spontaneous abortion were not systematically available in the anonymized database and could therefore not be adjusted for, constituting additional unmeasured confounders that should be captured in future prospective studies. Additionally, there was a lack of long-term offspring follow-up and unavailable embryonic ploidy data. Fourth, insulin resistance data were not systematically available in the clinical database, precluding stratification by metabolic phenotype, a relevant consideration given the known impact of insulin resistance on ovarian response and ART outcomes in PMOS. Fifth, metformin use and duration were not systematically recorded, and their potential influence on folliculogenesis and oocyte quality cannot be excluded. Additionally, as the study was conducted at a public center, where IVF treatments are restricted to women under 40 years old, the narrow age range limits the generalizability of our findings to older populations. Finally, our reliance on AMH and follicle count as the main predictors of ovarian response should be interpreted within the broader context of ovarian response assessment, since recent integrative frameworks combining static reserve markers with dynamic indices (e.g., follicular output ratio, follicle-to-oocyte index) have been proposed to refine individualized prediction of ovarian response to COS (41).

Therefore, this study’s results should be considered as hypothesis-generating, which require confirmation by larger randomized prospective multicenter studies to clarify whether the optimized follicular response observed with the addition of r-LH to r-FSH ultimately translates into higher CLBR, thereby allowing for a more robust personalization of ovarian stimulation protocols in women with PMOS.

## 5 Conclusion

This study showed that in women with PMOS, COS with r-LH/r-FSH enhanced follicular recruitment, as reflected by a significant increase in the number of follicles on the trigger day, but was not accompanied by significant differences in CPR, LBR or CLBR. Moreover, in women with PMOS, the positive effect of r-LH/r-FSH on follicular counts observed was independent of baseline AMH levels, supporting a potential role of r-LH in modulating ovarian response during COS in this specific population that warrants further investigation. However, based on the available evidence, in women with PMOS, routine use of r-LH during COS cannot be recommended as these findings are only hypothesis-generating, and require confirmation by larger, prospective randomized studies to determine whether the enhanced follicular response translates into improved CLBR in specific PMOS subpopulations.

## Data Availability

The datasets generated and/or analyzed during the current study are not publicly available due to institutional and ethical restrictions related to the use of patient-level clinical data. Requests to access the datasets should be directed to Emídio Vale-Fernandes, emidiovalefernandes.pma@ulssa.min-saude.pt.
